# Transcriptome comparisons detect new genes associated with apoptosis of cattle and buffaloes preantral follicles

**DOI:** 10.1186/s43141-021-00253-9

**Published:** 2021-10-08

**Authors:** Khairy Mohamed Zoheir, Ahmed Mohamed Darwish, Yang Liguo, Abdelkader E. Ashour

**Affiliations:** 1 Cell Biology Department, National Research Centre, Dokki, 12622 Egypt; 2 Key Laboratory of Agricultural Animal Genetics, Breeding and Reproduction of Ministry of Education, College of Animal Science and Technology, Huazhong Agricultural University, Wuhan, People’s Republic of China; 3 Department of Basic Medical Sciences, Kulliyyah of Medicine, International Islamic University Malaysia, 25200 Kuantan, Pahang Darul Makmur Malaysia

**Keywords:** Atresia, Preantral follicles, Gene expression, Apoptosis, Cattle, Buffalo

## Abstract

**Background:**

To develop new breeding technology to improve the breeding ability of bovine, it is the development trend to find the main reason for the occurrence of atresia in these organisms. Transcriptomes of small (100–120 μm) and large (200–220 μm) preantral follicles from cattle and buffalo ovaries were evaluated in vivo and in vitro to understand the transcriptional modulation in preantral follicles that leads to the phenomenon of atresia.

**Methods:**

The preantral follicles were checked as dead, damage, or live follicles in vivo and in vitro by using trypan blue then bisbenzimide and propidium iodine. Transcriptomes of small (100–120 μm) and large (200–220 μm) preantral follicles of cattle and buffalo were evaluated in vivo and in vitro by microarray and RT-PCR. Healthy preantral follicles were selected based on staining results, and then RNA was extracted from them.

**Results:**

The viability percentage of preantral follicles in cattle was higher (26.7% and 20%) than buffalo (10%) in vivo and in vitro, respectively. According to the microarray data analysis for cattle preantral follicles, only eleven genes were detected corresponding to five upregulated and six downregulated in large size (200–220 μm) compared to small (100–120 μm) size preantral follicles, while in buffalo, 171 genes were detected (92 upregulated and 79 downregulated) in large size compared to small preantral follicle size. The results of RT-PCR of the selected genes (FASTKD1, BAG2, RHOB, AGTR2, MEF2C, BCL10, G2E3, TM2D1, IGF-I, IGFBP3, PRDX3, and TRIAP1) validated the microarray results. In conclusion, the data of gene expression showed significant differences between small and large sizes in both buffalo and cattle preantral follicles.

**Conclusion:**

Apoptotic genes were upregulated in the large preantral follicle compared with the small preantral follicles. Moreover, the expression level of these apoptotic genes was significantly upregulated in buffalo than in the cattle. Most of these genes were significantly upregulated in the large buffalo preantral follicle compared with the small size. However, anti-apoptotic genes were upregulated in large cattle preantral follicle and downregulated in large buffalo preantral follicle.

## Background

Buffalo and cattle receive more attention than the other livestock species despite their importance as a potential source of meat and milk, particularly in developing countries.

The oocyte quality is the direct and most important factor for fertilization to develop an animal. Therefore, it is meaningful to explore the mechanisms of flucogenesis and oogenesis quality trait formation. Follicle and oocyte quality is one of the major challenges which the buffalo industry faces. Buffalo has fewer primordial follicles than bovine species (10,000–19,000 vs 150,000), smaller antral follicles, and a higher prevalence of atresia (82–92%) ([1]). The number of viable follicles and oocytes in newborn calves’ ovaries is ranging from 10,000 to 350,000 at birth, and in 12-month-old heifers, from 1920 to 40,960 ([2]). The neonatal bovine ovary contains many primordial follicles, but only about 0.1 percent of these follicles will mature and ovulate during a cow's reproductive development ([3]). Most follicles in the mammalian ovary die of atresia, and only a small number of follicles are selected for ovulation. Although follicle atresia happens during female reproductive life, the exact mechanism behind their extensive large cell death is indefinite. Several investigations suggested that follicle atresia is related to DNA fragmentation of the cell, an important biochemical marker of apoptosis [4]. Apoptosis has morphologic and biochemical characteristics and it is one of the types of programmed cell death [5]. The apoptotic events were induced by several signaling pathways [6]. One of the most important apoptotic pathways is the mitochondria-dependent pathway. Cytochrome c is speedily released from the mitochondria into the cytosol after exposing the cells to apoptotic stimuli [7]. Several genes, such as protein 53 (p53) [8], Bcl-2-associated X protein (Bax) [9], and B cell lymphoma 2 (Bcl-2) [7], are associated with the release of cytochrome c, either positively or negatively. The Liberation of Cytochrome c activates the cell death protease and caspase-3 release and thus can be fatal for cells [10].

Key post-transcriptional regulators like Fas-activated serine/threonine kinase (FASTK) family proteins appeared as mitochondrial gene expression and have altered function in the regulation of mitochondrial RNA [11]. G2/M phase-specific E3 **(**G2E3) extremely regulated at both levels of transcription and post-translation, plays an important role in early embryonic development and becomes downregulated in response to DNA damage, as murine G2E3-deficient blastula suffers massive apoptosis [12]. Ras Homolog Family Member B (RhoB) gene regulates many cellular processes such as gene transcription and cell cycle progression [13]. Insulin-like growth factors (IGFs) regulate follicular development and granulosa cell apoptosis and thus can block apoptosis enhanced by dexamethasone in many cell types (Armstrong et al. 2001).

To investigate the difference in apoptosis and its relationship with atresia between small and large healthy preantral follicles in both buffalo and cattle, we used a more ultimate procedure to determine changes in gene expression using the microarray assay. This investigation was designed to study the association of cell death program genes with preantral follicle apoptosis.

## Methods

### Chemicals

All the chemicals and antibiotics used in this study were purchased from Sigma-Aldrich Company, Ltd., Germany.

### Animals

We used ovaries from both buffalo and cattle slaughtered at an Islamic slaughterhouse in Wuhan, China. We removed all ovaries from the animals within a few minutes after slaughter.

### Isolation of preantral follicles

Twenty ovaries were collected from both buffalo (*Bubalus bubalis*) and cattle (*Bos taurus*), and then were placed in 10 bags and were kept in saline solution (0.9% NaCl) with penicillin (200 IU/mL) and streptomycin (200 μg/mL) at 30–35 °C.

According to Khairy et al. [14], we isolated different size of both buffalo and cattle preantral follicles as follows: sections of the ovarian cortex were excised by scalpel and placed into a tissue chopper adjusted to produce 500 μm sections. These small to minute segments were placed in Dulbecco’s phosphate-buffered solution supplemented with 36 μg mL^−1^ sodium pyruvate, 1 μg mL^−1^ glucose, and 3 mg mL^−1^ BSA. To remove all large fragments and debris, samples were filtered by a 500-μm filter and washed several times, then filtered by a 38-μm filter to exclude blood cells and other fine particles. The cells on the 38-μm filter were washed and collected in an embryo searching dish (100 × 20 mm, Falcon).

### In vivo experiment

The freshly isolated follicles were examined under an inverted microscope; the morphologically normal follicles were selected (healthy appearing, spherical with one or more compact layers of granulosa cells around the oocyte with an intact basal membrane, with no apparent sign of necrosis and no antrum).

Under an inverted microscope, two different sizes small (100–120 μm) and large (200–220 μm) of fresh healthy preantral follicles (*n*=100) from cattle and buffalo were selected.

### In vitro experiment

Only healthy preantral follicles (80–100 μm) were selected and cultured individually in droplets (*n*=100) of TCM-199 (20 μL) plus Follicle Stimulating Hormone (FSH) (100 ng/mL) and EGF (100 ng/mL) + epidermal growth factor (EGF) (100 ng mL^−1^). Then, it was supplemented with 10% NCS (Newborn Calf Serum, Gibco), 0.23 mM sodium pyruvate, 75 mg/ml of streptomycin, 100 mIU/ml of penicillin, 1% ITS (Insulin Transferring Selenium; Gibco), and 2.2 g/L sodium bicarbonate covered sterile mineral oil in falcon culture dishes at 38.5 °C, 100% humidity, and 5% CO_2_ for up to 25 days. To avoid contamination, the medium was changed with fresh medium every 3 days. At the end of the in vitro experiment, the diameter of follicles was measured by a camera of inverted microscope supported by diameter in its lenses, and then selected the following two sizes of follicles: 100–120 and 200–220 μm [14].

### Viability screening

To monitor the morphological deformity or degeneration, half of the preantral follicles from both in vivo and in vitro experiments were stained with Trypan blue to categorize their viability on the basis of the degree of dye exclusion. Unstained follicles were classified as viable and fully stained follicles as dead. Follicles with medium staining were regarded as damaged. And the rest of the preantral follicles were double-stained by bisbenzimide (H 33342) plus propidium iodine and compared with fresh controls to examine the morphological aspects such as the presence of an intact follicle membrane, a flattened or cuboidal granulosa cell layer, and presence of a nucleus. Double staining helped us to analyze the proportions of live and dead granulosa cells and also the nuclear components.

### Total RNA extraction from preantral follicles

Only healthy buffalo/cattle preantral follicles (100–150 preantral follicles) from every size were suspended and collected by low-speed centrifugation (triple samples for every size); then we added 300 μl lysis/binding buffer shake or repeatedly beat, so that the cells are lysed.

We used an isolation kit which was purchased from Applied Biosystem AM1556 to extract the total RNA. The concentration and quality of RNA were measured by Nanodrop (Thermo). Three replicates of each RNA sample for every size were processed for hybridization to Bovine Affymetrix Genome Array. Other parts of the same RNA samples were used for qPCR analysis for some genes to confirm microarray data.

### Microarray analysis

According to the manufacturer’s standard protocols, RNA of small and large preantral follicles samples were labeled, hybridized, and scanned. The obtained raw data from array images analysis by Affymetrix GeneChip Command Console (version 4.0, Affymetrix) was analyzed by Genesrping software. Differentially expressed genes were identified through fold change. Afterward, GO analysis and KEGG analysis were performed to detect the roles of these differential mRNAs.

### Real-time polymerase chain reaction

RNA was reverse transcribed into cDNA according to kit instruction of cDNA kit-Applied Biosystems (Catalog # 4374967) and purchased from Shanghi Sangon Biological Engineering Technology and Services Co., Ltd., which was then subjected to PCR amplification in the ABI Prism 7500 System (Applied Biosystems). Briefly, 1.5 μg of total RNA from each sample was added to a mixture of 2.0 μl of 10× reverse transcriptase buffer, 0.8 μl of 25× dNTP mix (100 mM), 2.0 μl of 10× reverse transcriptase random primers, 1.0 μl of multi-scribe reverse transcriptase, and 3.2 μl of nuclease-free water. The final reaction mixture was held at 25 °C for 10 min, then heated to 37 °C for 120 min and 85 °C for 5 s, and, finally, cooled to 4 °C.

Quantitative analysis of target gene mRNA expression was performed via RT-PCR by subjecting the cDNA obtained from the above preparation methods to PCR amplification in 96-well optical reaction plates using the ABI Prism 7500 System (Applied Bio systems). The 25-μl reaction mixture contained 0.1 μl of 10 μM forward primers and 0.1 μl of 10 μM reverse primers (40 μM final concentration of each primer), 12.5 μl of SYBR Green Universal Mastermix, 11.05 μl of nuclease-free water, and 1.25 μl of the cDNA sample. The primers used in these assays were designed from PubMed (https://www.ncbi.nlm.nih.gov/tools/primer-blast/index.cgi?LINK_LOC=BlastHome) and other databases and then were synthesized by Shanghi Sangon Biological Engineering Technology and Services Co., Ltd. which are listed in Table 1. Assay controls were incorporated into the same plate, which consisted of no template controls to test for contaminations of any of the assay reagents. The real-time PCR data were analyzed using the relative gene expression (i.e., ΔΔCT) method, as described in Applied Biosystems User Bulletin No. 2. Briefly, the data are presented as the fold change in gene expression normalized to an endogenous reference gene (GAPDH) and relative to a calibrator.

**Table 1:** Primer sequences

Gene symbol	Accession No.	Primer sequence (5′->3′)	Product size	Annealing
GAPDH	NM_001034034.2	F: GAGCTTGACAAAGTGGTCGTTGAG R: CCAACGTGTCTGTTGTGGATCTGA	218	55
FASTKD1	NM_001102031.1	F: GGTACAGAGTTCGGAGGCTT R: AGTGCACATCCCACTTGCTT	386	55
BAG2	NM_001034264.1	F; AACCGTCTGATGGGACGAAC R: CTGTGTGGCTGCTTTAGGGA	779	55
RHOB	NM_001077922.1	F: GCCAACAAGAAAGACCTGCG R: ATAACACCCCATCCTCCCCA	763	55
AGTR2	XM_024988817.1	F: ACTCGAACAACGAAAGGTGT R: AGCTGTTGGTGAATCCCAGG	983	55
TM2D1	NM_001079593.2	F: CGATTGCTCCCGATACTGCT R: TCAAGGAGGCCCAAATCTGT	629	55
MEF2A	NM_001083638.2	F: ACAGCCCAGACCCTGATACT R: AGCATTCCTGGCGAGTTGAA	763	55
BCL10	NM_001078028.1	F: AGCCTTTTCCTGATGGAGCC R: ACAGCACGTGATCGTAAGGG	313	55
G2E3	NM_001038671.2	F: AGACACGACTGAGAAGCTAATAC R: TCTTCGGACGTCACAATGCT	754	55
PRDX3	NM_174432.2	F: TCCTCTACGTCTCTGTCGGG R: TGCTTGATCGGAACCAGACC	198	55
TRIAP1	NM_001079776.1	F: GCCTGACCTTGAGCCATTCT R: TGTTGTGCTGGGAACCCTTT	131	55
IGF-I	NM_001077828.1	F: TGTGATTTCTTGAAGCAGGTGAAG R: TTCATTGGGGGAAATGCCCA	556	55
IGFBP3	NM_174556.1	F: AGCGCAGCAGCTATTCCAA R: TGCTGTGGTCTTCTTCCGAC	565	55

### Statistical analysis

Each value indicates the mean ± SE of 100 preantral follicles per group. Statistical analysis was performed using one-way ANOVA followed by the Tukey–Kramer post-test (SPSS program version 17).

## Results

**Fig. 1:**
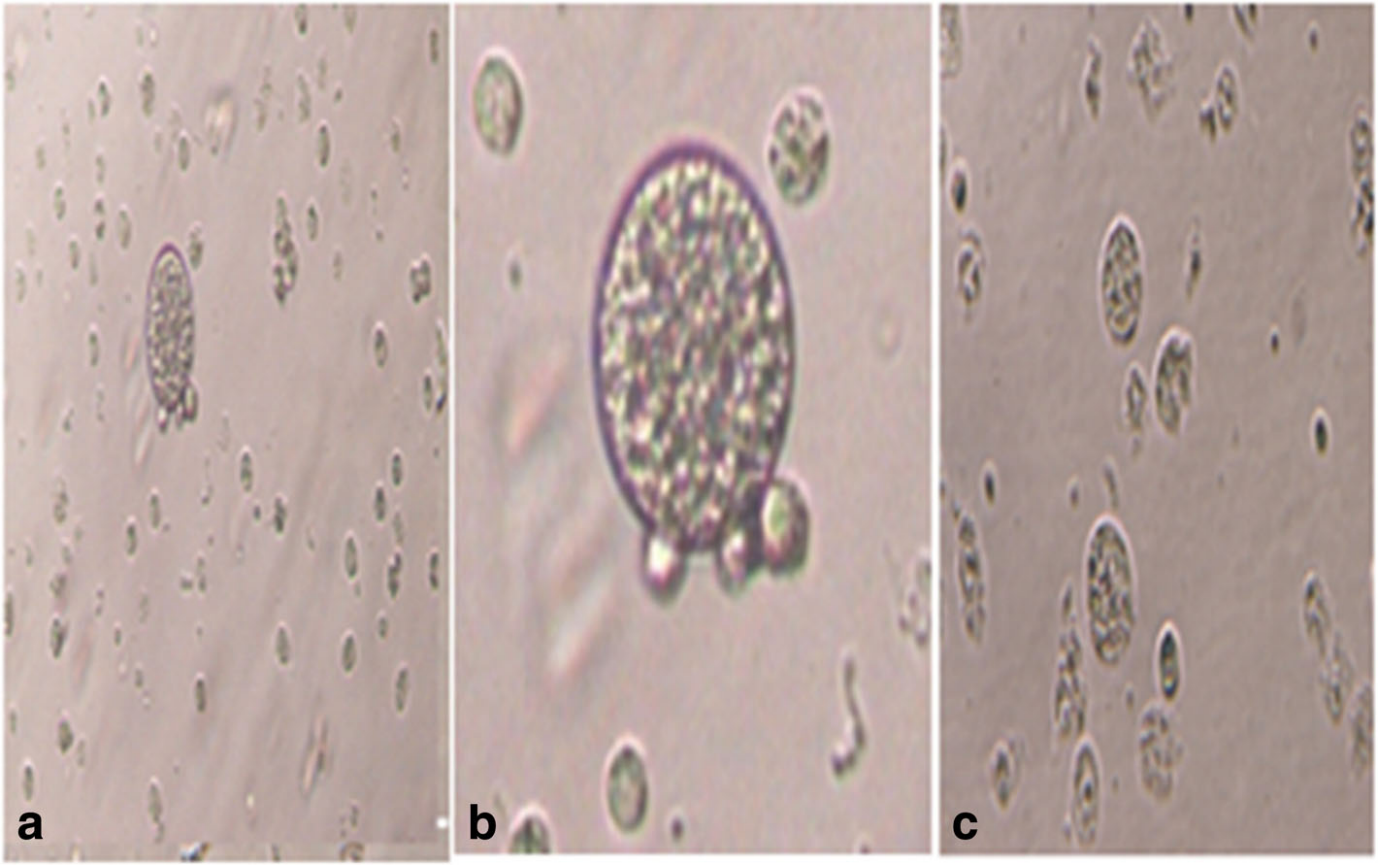
Showing different sizes of bovine and buffalo preantral follicles. **a** and **b** represent healthy preantral follicles and **c** represent non-healthy (degenerated) preantral follicles

Based on the staining results, we found that the ratio of healthy preantral follicles in cattle was higher (26.7% and 20%) than buffalo (10% and 10%) in vivo and in vitro experiments, respectively (Table 2), which may explain why the yield of buffalo oocytes is lower than cattle oocytes per one ovary. After normalizing the microarray data for cattle preantral follicles, eleven genes were detected in cattle follicles (five upregulated and six downregulated) in large size compared to small size follicles. However, in buffalo follicles, 171 genes were detected (92 upregulated and 79 downregulated) in large size compared to small size follicles. Fold changes of the selected genes in cattle and buffalo were summarized in Table 3. The small and large sizes of bovine and buffalo preantral follicles were shown in Fig. 1.

**Table 2:** The percentage of viability of preantral follicles

	No. of isolated preantral follicles	Buffalo			Cattle		
		Dead	Damage	Live (healthy)	Dead	Damage	Live (healthy)
In vivo isolated preantral follicles	3000	1200 (40%)	1500 (50%)	300 (10%)	1000 (33.3%)	1200 (40%)	800 (26.7%)
In vitro isolated preantral follicles	1000	500 (50%)	400 (40%)	100 (10%)	400 (40%)	400 (40%)	200 (20%)

**Table 3:** Microarray data show description and fold changes for some genes of study

Probe set	Gene symbol	Gene description	Gene bank	Gene loc.	***P*** value	Large size	Small size	Fold change
**Cattle**								
Bt.8382.1.S1_at	RHOB	Ras homolog gene family, member B	NM_001077922	Ch.11	0.023	7.09	6.06	+ 1.03
Bt.27353.1.A1_at	AGTR2	Angiotensin II receptor, type 2	XM_001249373 XM_002699453	Ch.X	0.037	4.1	3.09	+ 1.02
Bt.26582.2.S1_at	TM2D1	TM2 domain containing 1	NM_001079593	Ch.3	7.32	6.09	4.7	+ 1.3
Bt.27103.1.A1_at	FASTKD1	FAST kinase domains 1	NM_001102031	Ch.2	0.39	3.75	5.07	− 1.32
Bt.10885.2.S1_at	BAG2	BCL2-associated athanogene 2	NM_001034264	Ch.23	0.003	6.76	7.83	− 1.06
Bt.12750.1.S1_at	IGF1	Insulin-like growth factor 1	NM_001077828	Ch5	0.046	5.39	4.3	+ 1.08
**Buffalo**								
Bt.26582.2.S1_at	TM2D1	TM2 domain containing 1	NM_001079593	Ch.3	7.3	5.6	3.9	+ 1.68
Bt.5092.1.S1_at	PRDX3	Methylthioadenosine phosphorylase peroxiredoxin 3	NM_174432 XM_001251562 XM_002689506	Ch.24	2.4	10.75	7.73	+ 3.02
Bt.25343.1.S1_at	TRIAP1	TP53 regulated inhibitor of apoptosis 1	NM_001079776	Ch.17	2.4	10.16	8.79	+ 1.36
Bt.10885.2.S1_at	BAG2	BCL2-associated athanogene 2	NM_001034264	Ch.23	0.003	7.36	9.44	− 2.07
Bt.16150.1.A1_at	MEF2C	Myocyte enhancer factor 2C	NM_001046113	Ch.7	0.08	6.04	7.6	− 1.6
Bt.19656.2.A1_at	BCL10	B cell CLL/lymphoma 10	NM_001078028	Ch.3	0.056	4.7	5.7	− 1.08
Bt.22493.1.S1_at	G2E3	G2/M-phase specific E3 ubiquitin protein ligase	NM_001038671	Ch.21	0.003	4.7	5.9	− 1.19
Bt.422.1.S1_at	IGFBP3	Insulin-like growth factor binding protein 3	NM_174556	Ch4	2.4	7.6	9.8	− 2.18

### Gene expression results

**Fig. 2:**
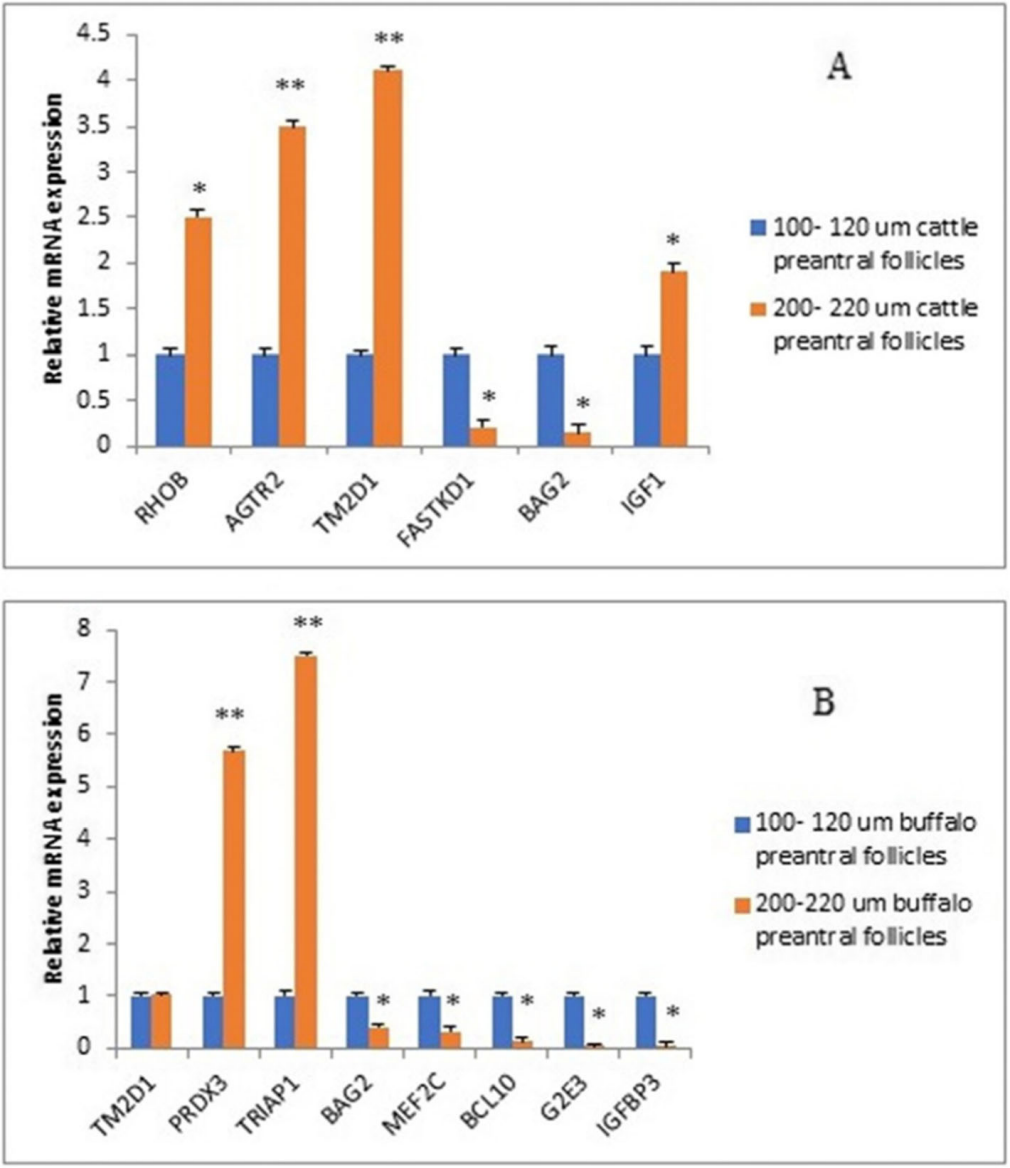
The expression values of apoptotic genes in preantral follicles for large and small size in both buffalo and cattle

To confirm the data from microarray, we selected only 12 genes to assess their mRNA expression in two types (small and large) of preantral follicles: four genes (FASTKD1, IGF-I, RHOB, and AGTR2) in cattle only, two genes (BAG2 and TM2D1) in both cattle and buffalo, and six genes (MEF2C, BCL10, G2E3, IGFBP3, PRDX3, and TRIAP1) in buffalo only. We found that FASTKD1 and BAG2 were significantly downregulated in large size (200–220 μm) cattle preantral follicles when compared to the small size (100–120 μm), while RHOB, AGTR2, IGF-I, and TM2D1 were significantly upregulated (Fig. 2A). In buffalo, we found that transcript levels of BAG2, MEF2C, BCL10, IGFBP3, and G2E3 were significantly downregulated in large size when compared to small size follicles but both PRDX3 and TRIAP1 mRNA expression were significantly upregulated in large size when compared to small sizes (Fig. 2B).

## Discussion

Buffalo and cattle are an important livestock resource playing a crucial role in the agriculture economy of several countries of the Mediterranean regions including Egypt and East Asia like China. Complete list of flucogenesis and oogenesis and their roles in bovine’s fertility are not known. Little is known about the molecular changes associated with the development of small to mature follicles. In many organisms, apoptosis is a normal part of development. Apoptosis is a well-thought-out vital component of several processes including change in gene expression, cell turnover, and the embryonic development. Owing to the complexities in the isolation of preantral follicles, the nature of these cell’s death remains vaguely understood [5].

The apoptosis rate of the granulosa cells tends to increase during follicular development. Apoptosis is an ongoing process in ovarian preantral follicles and is most likely related to atresia [15]. We have focused our study on the assessment of expression of apoptosis-related genes in two stages of buffalo and cattle preantral follicle development, namely small and large preantral follicles which directly lead to follicular maturation. In cattle, we detected variances in an expression of only 11 apoptosis-related genes by microarray analysis, six downregulated (FASTKD1, BAG2, PTCH1, DBH, SHH, PTK2), and five upregulated transcripts in large follicles (LCK, RHOB, AGTR2, TM2D1, IGF1) when compared to small preantral follicles, while in buffalo, we found variances in an expression of 171 apoptosis-related genes, 92 upregulated, and 79 downregulated transcripts in large preantral follicles when compared to small preantral follicles size.

FASTKD1 was shown to protect cells from oxidative stress-induced cell death and to suppress autophagy, whereas downregulation of FASTKD1 sensitized cells to cell death mechanisms [16]. Bcl-2-associated athanogene (BAG) family proteins interact with various partners included in modifying the proliferation/death balance, including the anti-apoptotic Bcl-2, and enhance its anti-apoptotic effects [17]. Our results were in agreement with Yang et al. [18] as we have detected significant differences between large and small follicles in both cattle and buffalo. IGFs are considered one of the inhibitor cytokines for apoptosis and play a vital role in regulating cellular homeostasis [19]. Healthy follicles had levels of IGF-I significantly higher than slightly atretic and atretic follicles [20]. Also, Ginther et al. [21] provide that the level of IGF-I increased in the largest follicles of bovine. Besides, IGFs had been correlated with changes in negative and positive regulators of the Bcl-2 family and alteration of Bax and Bcl2 levels [22]. The present results of microarray and RT-PCR show that IGF-I was upregulated in large preantral follicles of cattle compared with small size follicles while IGFBP3 was downregulated in large preantral follicles of buffalo.

MEF2C acts as an anti-apoptotic factor and keeps the transplanted cells alive after differentiation in embryonic stem cells [23]. Our microarray and qPCR data detected changes in the mRNA level in large preantral follicles when compared to the small preantral follicles. AGTR2 is transcribed from an X-linked gene and enters in several signaling cascades influencing neuronal differentiation, cell proliferation, growth inhibition, and induction of apoptosis [24]. G2E3 gene plays an effective role in early embryonic development. Also, it acts as a regulator for cell death because it causes cellular apoptosis when reduced [12]. PRDX3 gene is in the control of cell proliferation and apoptosis as was cited by Rhee and Woo [25]. Downregulation of PRDX3 enhances cisplatin-induced ovarian cancer cell apoptosis [26]. TR1AP1 alters the transcriptional activity of the genes correlated to cell death, so it was looked upon as initiating apoptosis [27].

The present study showed that the viability of preantral follicles in cattle was higher than buffalo, and damaged or dead preantral follicles in buffalo were higher than cattle. The intrinsic species-specific lower number of primordial and antral follicles in buffalo compared to cattle were investigated which underlines the difficulty of the great variability in follicular recruitment in this animal [28, 29]. It has been reported that buffalo has a smaller number of primordial follicles than bovine species, smaller antral follicles, and a higher incidence of atresia [30]. It is possible to think that animals with low follicle count such as buffalo have lower fertility than cattle [31], and this change in fertility between these two species is related to changes in the transcriptome.

On the other hand, the present results of RT-PCR showed that Rhob, TM2D1, and IGFI which are considering as anti-apoptotic genes were upregulated in large cattle preantral follicle while applied anti-apoptotic genes (TM2D1, G2E3, MEF2, and IGFBP3) were downregulated in large buffalo preantral follicle.

Although we found out that many genes were either down- or upregulated more in buffalo preantral follicles than in cattle leading to increasing apoptosis and supporting our previous suggestion, we found other few genes for which anti-apoptotic genes were upregulated more in buffalo than in cattle. These results conflicted with a lot of genes which supported the hypothesis that apoptosis were more in buffalo follicles than in cattle. The presence of several genes opposing this content was clear and explicit.

FASTKD1, for example, which is considered as anti-apoptotic gene (antiapoptotic, prosurvival), was significantly downregulated in large cattle preantral follicles. RHOB (pro-apoptotic) gene also increased only in large cattle preantral follicles when compared to small preantral follicles This conflict in transcriptome related to the apoptotic process opened the door for new studies to focus on the dynamics of these genes working within a single cell by using single cell-RNA sequencing.

## Conclusion

The mechanism of buffalo follicular development is different from that of cattle, which is the main reason for the lack of oestrus, irregular ovulation, and low conception rate in estrus. The staining results consent with microarray, and RT-PCR results showed that the percentage of healthy cattle preantral follicles was more than buffalo. Microarray results showed that many genes related to apoptosis in buffalo (171 genes) were more than cattle (11 genes). RT-PCR data indicated that most of the apoptosis genes were upregulated in large preantral follicles, and anti-apoptosis genes were downregulated in buffalo. In contrast, the apoptotic genes were downregulated in large preantral follicles, and anti-apoptosis genes were upregulated in cattle which mean that preantral follicles with successful maturation contain anti-apoptosis genes or downregulated apoptotic genes. Also, these new data for transcriptome may open a new window for confirmation that cattles are more fecund than buffalo.

## Data Availability

Data is available for all.
